# Supplementary material for: Bacitracin and Rutin Regulate Tissue Factor Production in Inflammatory Monocytes and Acute Myeloid Leukemia Blasts
Source: Cancers (Basel). 2021 Aug 4;13(16):3941. doi: 10.3390/cancers13163941 (PMC8393688; doi:10.3390/cancers13163941)
Supplement: Supplementary file 1 [file cancers-13-03941-s001.zip › cancers-1299730-supplementary.pdf]

*Article*

# Bacitracin and rutin regulate tissue factor production in inflammatory monocytes and acute myeloid leukemia blasts

**Lennart Beckmann [1,†], Christina C. Rolling [1,2,†], Minna Voigtländer [1], Jonathan Mäder [1], Felix Klingler [1], Anita Schulenkorf [1], Carina Lehr [1], Carsten Bokemeyer [1], Wolfram Ruf [3,4] and Florian Langer [1,*]**

## Supplementary Table and Figure Legends

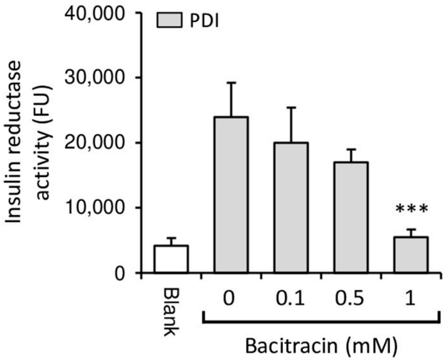


**Figure S1.** Bacitracin dose-dependently inhibits protein disulfide isomerase (PDI) insulin reductase activity. human recombinant PDI was incubated with increasing concentrations of bacitracin (0–1 mM) for 30 minbefore insulin reductase activity was measured using a fluorogenic assay (mean ± SD, n = 3). P-value is according to Tukey´s post hoc test (***, $p < 0.001$).

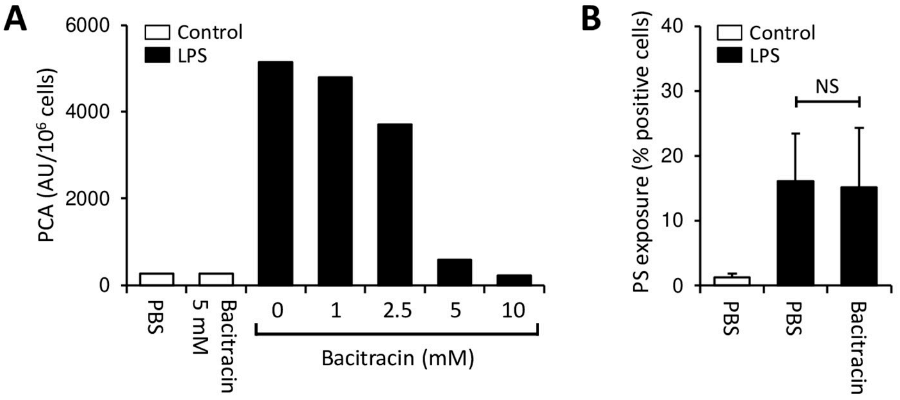


**Figure S2.** Co-incubation with bacitracin dose-dependently inhibits lipopolysaccharide (LPS)-induced tissue factor (TF) procoagulant activity (PCA) in peripheral blood mononuclear cells (PBMCs) but has no effect on phosphatidylserine (PS) membrane exposure. **(A)** LPS-treated PBMCs were analyzed for PCA by one-stage clotting assay following co-incubation with increasing concentrations of bacitracin (0–10 mM). A representative experiment is shown. **(B)** PBMCs were incubated with 100 ng/mL LPS in the presence or absence of 5 mM bacitracin for 4 h at 37 °C before exposure of PS was measured on CD14-positive monocytes by flow cytometry (mean ± SD, n = 4). P-values are according to Tukey´s post hoc test (NS, not significant).

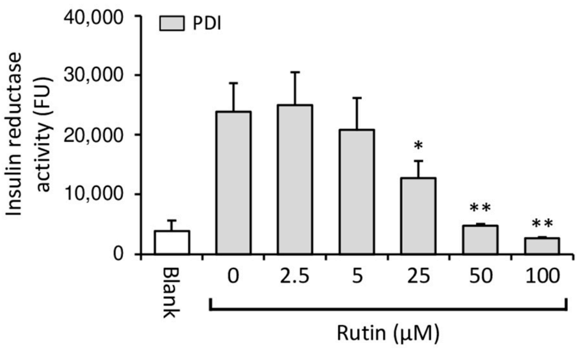


**Figure S3.** Rutin inhibits PDI insulin reductase activity in a concentration-dependent manner. Following incubation of human recombinant PDI with increasing concentrations of rutin (0–100 µM) for 30 min, insulin reductase activity was measured using a fluorogenic assay (mean ± SD, n = 3). P-values are according to Tukey´s post hoc test (*, $p < 0.05$; **, $p < 0.01$).

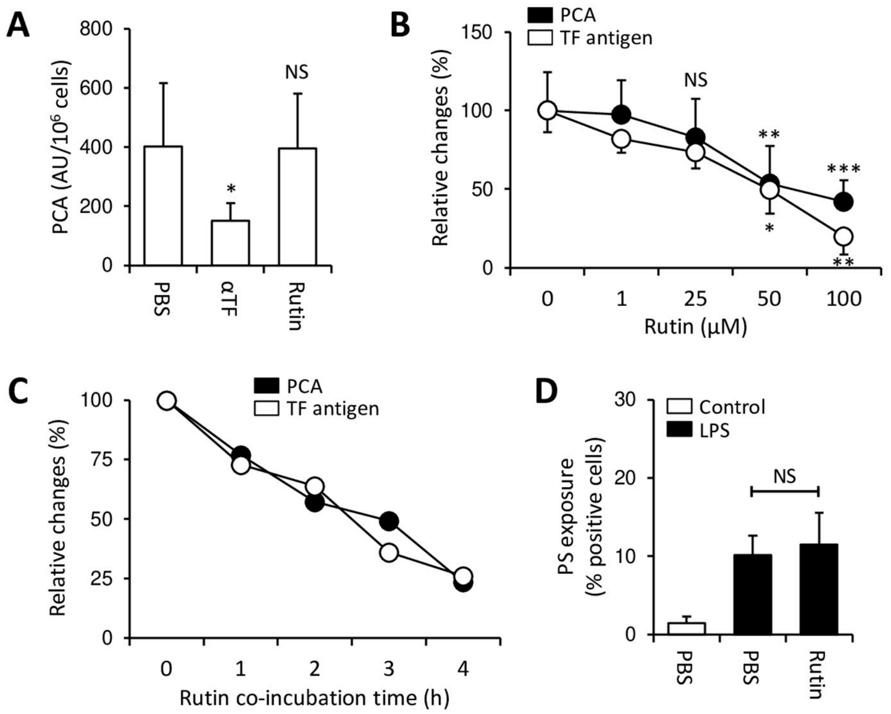


**Figure S4.** Rutin inhibits LPS-induced TF production in PBMCs in a time- and concentration-dependent manner. **(A)** PBMCs were incubated in the presence or absence of rutin (100 μM) for 4 h at 37 °C and subsequently analyzed for TF PCA by single-stage clotting assay (mean ± SD, n = 7). **(B)** PBMCs were co-stimulated with LPS (100 ng/mL) and increasing concentrations of rutin (0–100 μM) for 4 h at 37 °C before being analyzed for TF PCA (closed dots) or monocyte TF antigen expression (open dots) (mean ± SD, n = 3). **(C)** PBMCs were stimulated with LPS (100 ng/mL) for 4 h, while rutin (100 μM) was added for indicated durations towards the end of the LPS incubation period. Afterwards, TF PCA (closed bars) and monocyte TF antigen expression (open bars) were recorded. A representative experiment is shown. **(D)** Following stimulation of PBMCs with LPS (100 ng/mL) in the presence or absence of rutin (100 μM) for 4 h at 37 °C, PS exposure on CD14-positive monocytes was measured by flow cytometry (mean ± SD, n = 5). P-values are according to Tukey´s post hoc test (NS, not significant; *, $p < 0.05$; **, $p < 0.01$; ***, $p < 0.001$).

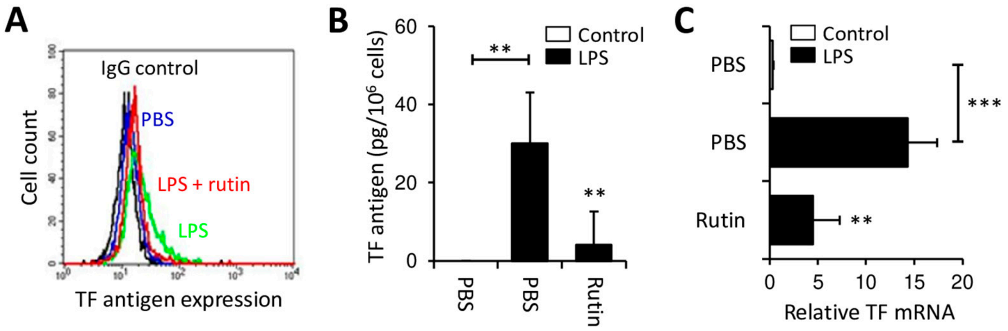


**Figure S5.** Rutin inhibits LPS-induced TF production by PBMCs in whole blood. **(A)** Monocyte TF antigen expression was analyzed by flow cytometry following stimulation of citrate-anticoagulated whole blood as indicated for 4 h at 37 °C. A representative histogram from 8 independent experiments is shown. **(B)** PBMCs were purified from stimulated whole blood by density gradient centrifugation and analyzed for total TF antigen by ELISA (mean ± SD, n = 5). **(C)** Following stimulation of citrate-anticoagulated whole blood for 2 h at 37 °C with 10 µg/mL LPS in the presence or absence of 100 µM rutin, PBMCs were purified and analyzed for TF mRNA by quantitative RT PCR (mean ± SD, n = 5). P-values are according to Tukey´s post hoc test (**, $p < 0.01$; ***, $p < 0.001$).

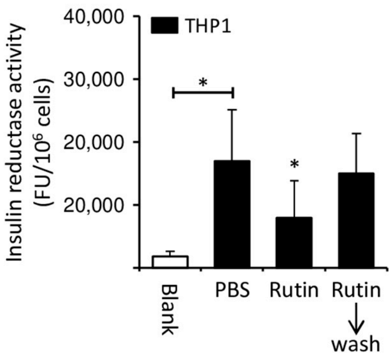


**Figure S6.** Short-time incubation with rutin reversibly inhibits insulin reductase activity of THP1 cells. THP1 cells were exposed to rutin (100 µM) for 30 minand analyzed for insulin reductase activity both before and after washing with PBS (mean ± SD, n = 3). P-values are according to Tukey´s post hoc test (NS, not significant; *, $p < 0.05$).

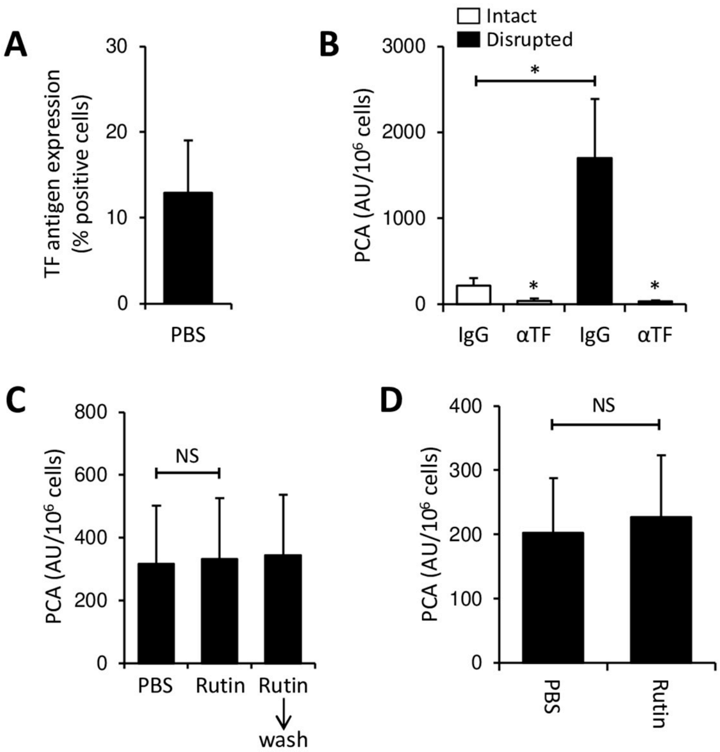


**Figure S7.** TF is predominantly cryptic on intact THP1 cells. **(A, B)** THP1 cells were analyzed for TF antigen expression by flow cytometry (mean ± SD, n = 7) **(A)** and for TF PCA by single-stage clotting assay both before and after physical disruption by repeated freeze-thawing (mean ± SD, n = 6) **(B)**. **(C)** THP1 cells were exposed to rutin (100 μM) for 30 minand analyzed for PCA both before and after washing with PBS (mean ± SD, n = 4). **(D)** THP1 cells were treated with rutin (100 μM) for 24 h at 37 °C and analyzed for PCA (mean ± SD, n = 7). P-values are according to Tukey´s post hoc test (NS, not significant; *, $p < 0.05$).

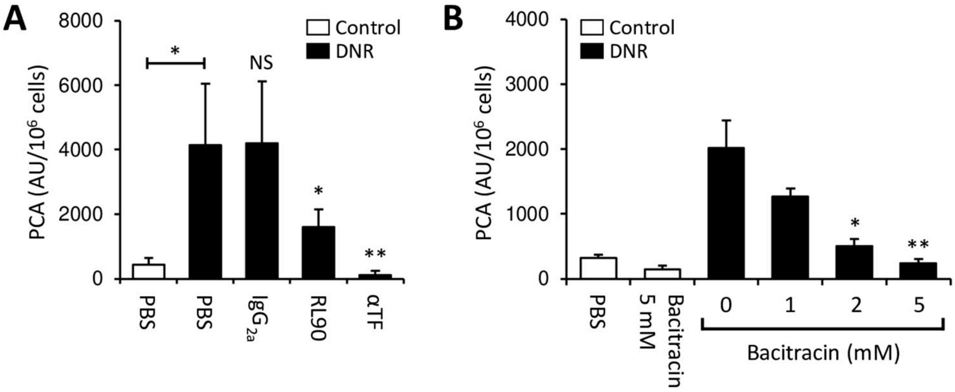


**Figure S8.** PDI inhibition on THP1 cells prevents activation of cryptic TF. **(A)** THP1 cells were treated with 1 µM DNR for 24 h at 37 °C in the presence of 50 µg/mL inhibitory PDI antibody RL90 or isotype control ($IgG_{2a}$) and subsequently analyzed for PCA by single-stage clotting assay (mean ± SD, n = 4). An inhibitory TF antibody (αTF) was added to DNR-treated cells prior to PCA measurement to demonstrate TF specificity. **(B)** U937 cells were treated with 1 µM DNR in the presence of increasing concentrations of bacitracin (0–5 mM) and subsequently analyzed for PCA by single-stage clotting assay (mean ± SD, n = 3). P-values are according to Tukey´s post hoc test (*, $p < 0.05$; **, $p < 0.01$).

**Table S1.** Demographic and clinico-pathological patient characteristics.

| | Reference range | Patient 1 Figure 5 | Patient 2 Figure 6A | Patient 3 Figure 6B |
|---|---|---|---|---|
| Age (years) | | 51 | 64 | 73 |
| Gender | | Female | Female | male |
| FAB subtype | | M2 | M5 | CMML/M5 |
| Molecular abnormalities | | *FLT3*-ITD | *FLT3*-ITD, NPM1 | n.a. |
| Cytogenetic abnormalities | | no | no | 50-51, XY, +5,+8,+15,+20, del(7)(q22), +mar24 |
| WHO classification | | AML NOS | AML with recurrent genetic abnormalities | AML with myelodysplasia-related changes |
| ELN classification | | adverse | intermediate | adverse |
| Bone marrow blasts (%) | | 64 | 73 | 30 |
| Leukocytes (1 x $10^9$/L) | 3.8–11.0 | 26.4 | 3.2 | 18.6 |
| Platelets (1 x $10^9$/L) | 150–400 | 60 | 172 | 40 |
| Hemoglobin (g/dL) | 12.3–15.3 | 8.0 | 9.5 | 11.3 |
| Prothrombin time (%) | 80–130 | 109 | 101 | 70 |
| Fibrinogen (g/L) | 1.8–4.0 | 3.1 | 3.6 | 2.4 |
| D-dimer (mg/L) | < 0.5 | n.a. | n.a. | 16.5 |
| LDH (U/L) | 84–246 | 746 | 214 | 943 |

Abbreviations are as follows: del, deletion; ELN, European Leukemia Net; FAB, French-American-British Classification; FLT3, FMS-like tyrosine kinase 3; ITD, internal tandem duplication; mar, marker chromosome; n.a., not analyzed; NOS, not other specified; NPM1, nucleophosmin 1
